# Canting of the occlusal plane: Perceptions of dental professionals and laypersons

**DOI:** 10.4317/medoral.18335

**Published:** 2013-03-25

**Authors:** Amparo Olivares, Ascensión Vicente, Carmen Jacobo, Sara M. Molina, Alicia Rodríguez, Luis A. Bravo

**Affiliations:** 1 Postgraduate student, Orthodontic Teaching Unit, Dental Clinic, University of Murcia, Murcia, Spain; 2 DDS,PhD. Contracted Doctor, Orthodontic Teaching Unit ,Dental Clinic, University of Murcia , Murcia, Spain; 3 MD,DDS,PhD., Senior Lecturer, Orthodontic Teaching Unit ,Dental Clinic, University of Murcia, Murcia, Spain

## Abstract

Objectives: To determine if canting of the occlusal plane influences esthetic evaluation of the smile among orthodontists, dentists and laypersons. Study Design: A frontal photo of a smile with 0º occlusal plane canting in relation to the bipupillary plane was modified using Adobe Photoshop C3 (Adobe Systems Inc, San José, California) to generate two images with occlusal plane inclinations of 2º and 4º. The three images were evaluated esthetically by orthodontists (n=40) general dentists (n=40) and laypersons (n=40). Each image was awarded a score as follows: 1=esthetically acceptable; 2=moderately acceptable; 3=esthetically unacceptable. Evaluators also placed the three images in order in preference. Data were analyzed using the Kruskal-Wallis (p<0.05) and the Mann-Whitney tests, applying the Bonferroni Correction (p<0.016). Results: No significant differences (p> 0.05) were found between the three groups for 0º and 2º cants (median for orthodontists=1; general dentists=1; laypersons=1). Orthodontists (median score=3) made evaluations of the image with 4º occlusal plane that were significantly different from general dentists (median=2) and laypersons (median=2). All three groups put the 0º image in first place in order of esthetic acceptability, the 2º image in second place and the 4º image in third place. Orthodontists placed the 0º image in first place with significantly greater frequency (p<0.016) than laypersons. Conclusions: Occlusal plane canting of 0º and 2º were evaluated as esthetically acceptable by the three groups. The 4º occlusal plane cant was evaluated more negatively by orthodontists than by general dentists and laypersons. All three groups placed the 0º image in first place of esthetic acceptability, 2º in second place and 4º in third. Orthodontists put the 0º image in first place with significantly greater frequency than laypersons.

** Key words:**Canting, perception, smile, orthodontics, dental esthetics.

## Introduction

Smile analysis and smile design have become a key element in orthodontics (1) but the subjective perception of beauty makes it difficult to establish concrete esthetic objectives for guiding diagnosis and treatment planning.

Various studies of facial features have been made with the aim of determining norms that might help orthodontists to evaluate facial characteristics (2-4), given that while the rules defining esthetics can be difficult to determine, it might be possible to form some general guidelines for optimizing dentofacial esthetics (5,6).

Occlusal plane canting is one characteristic that must be evaluated in any assessment of smile esthetics. It describes the vertical position of the teeth when the left and right sides are different and this is defined as the rotation upwards or downwards in the transversal plane of one side over the other. It can be observed both in the frontal plane and obliquely, whenever the lips are relaxed but most clearly in the smile.

Perceptions of esthetics vary from person to person and are influenced by gender, personal experience and social milieu (2,4). For the same reasons, there may be differences of opinion between the esthetic perceptions of laypeople and professionals (7). While Roden-Johnson et al. (8) and Pinho et al. (9) found that general dentists, orthodontists and laypersons evaluated features of the smile differently, other researchers such as Ioi et al. (10), Ritter et al. (11) and Martin et al. (12) have found that different groups classify the attractiveness of a smile in similar ways.

The objectives of the present study were to determine whether occlusal plane canting influences esthetic evaluations of the smile among orthodontists, general dentists and laypeople, as well as to find out if there is homogeneity of the criteria by which smile esthetics are assessed between professionals (orthodontist and general dentists) and lay people.

## Material and Methods

-Model selection and image manipulation

The Department of Dentistry’s digital archives (University of Murcia) were searched and a patient selected who had a smile with characteristics that fulfilled standard norms. The patient was called to the clinic and two photographs were taken with a digital camera (Canon EOS 450D, Madrid, Spain) with the head held in a natural position, one extraoral with the model smiling and another frontal intraoral photo. The patient gave informed consent for manipulating the images in this study.

**Figure 1:**
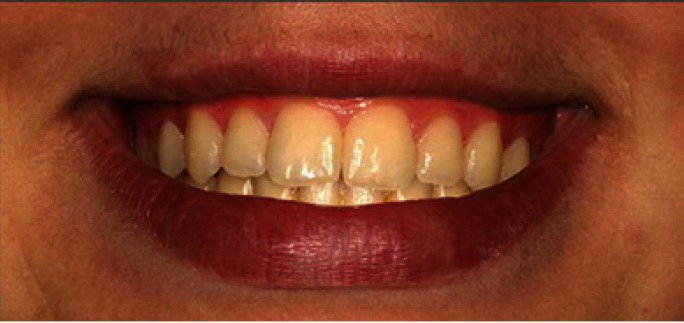
Photo of smile modified to create occlusal canting of 2º.

**Figure 2:**
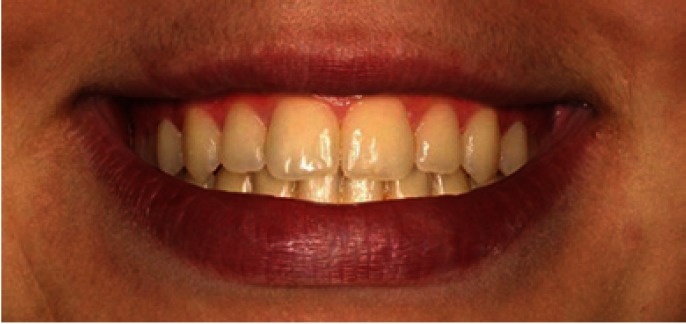
Photo of smile with 0º occlusal canting.

**Figure 3:**
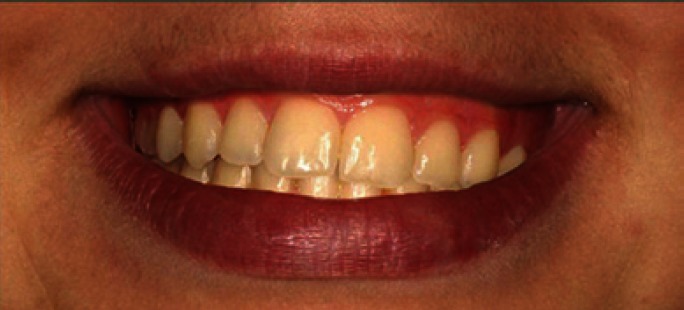
Photo of smile modified to create occlusal canting of 4º.

The extraoral photo provided the interpupillary line as a reference and from this, taking the frontal intraoral photo, three intraoral images were created, one with an occlusal plane cant of 2º (Fig. 1), one of 0º (Fig. 2), and one of 4º (Fig. 3).The images were trimmed down so that they framed the smile, showing only the tip of the nose and the mentolabial fold.

-Questionnaire and catalogue

A questionnaire and catalogue were designed including the set of smile images in color. The questionnaire gathered data on the subjects/evaluators: age, sex and profession. The catalogue contained four pages, with one image of the smile on each of three pages and the three images together on the fourth page.

The images were evaluated by the three groups of evaluators as 1=esthetically acceptable, 2=moderately acceptable, 3=esthetically unacceptable.

Each image was evaluated in less than 40 seconds.

-Evaluators

Evaluators consisted of 120 individuals (n= 60 men and n=60 women) from different cities in Spain, belonging to one of three groups: 40 orthodontists (20 men and 20 women) who had been in professional practice for over ten years; 40 general dentists (20 men and 20 women) who hade been in professional practice for over ten years; 40 laypersons (20 men and 20 women) aged between 40 and 50 years.

-Statistical Analysis

The presence of significant differences between the three groups of evaluators for individual assessment of each image and for order of preference were analyzed using the Kruskal-Wallis non-parametric test (p<0.05) and the Mann-Whitney test, applying the Bonferroni correction (p<0.016).

In order to evaluate the degree of agreement as to order of preference awarded to each image by professional (dentists and orthodontists) and laypersons, the Levene test between the standard deviations of the two groups was applied (p<0.05).

-Methodological error

Ten subjects from each group completed the questionnaire again after a two-week interval. The Wilcoxon Test for paired samples did not detect significant differences between image evaluations performed at these two different times (p>0.05). With regard to the order of preference, when data from the two time points were analyzed, orthodontists and dentists coincided across the two evaluation times in 100% of cases, while laypersons coincided in 80% of cases.

## Results

The Kruskal-Wallis test did not find significant differences in image evaluation between figure 1 (2º) and 2 (0º) (p= 0.97 and p=0.25 respectively). The Kruskal-Wallis test did find significant differences (p= 0.00) in the assessment of 3(4º), and the Mann-Whitney test detected difference between orthodontists and the other two groups (dentists p= 0.007, laypersons p= 0.000) ( Table 1).

**Table 1:**
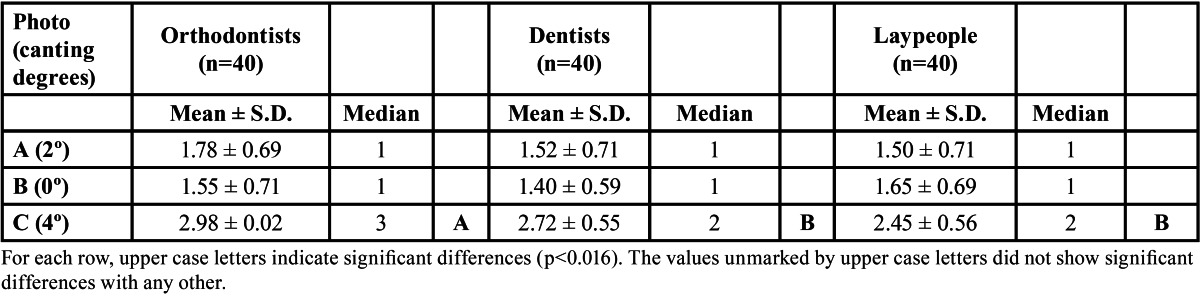
Mean, standard deviation (S.D.) and median for attractiveness scores allotted to images with different degrees of canting.

4 shows the order of preference in which the three evaluation groups placed each image.

**Figure 4:**
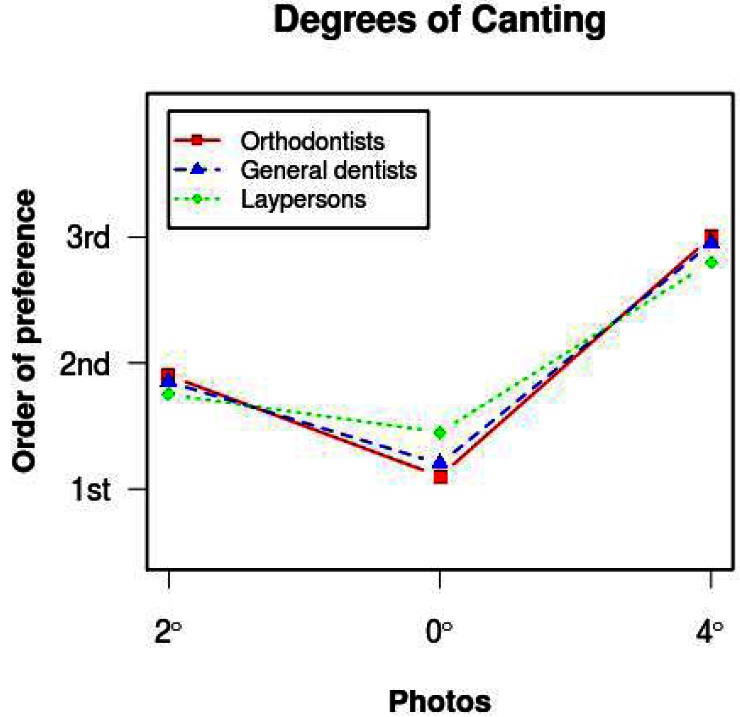
Order of preference allotted to each image by the three groups of evaluators.

The Kruskal Wallis test did not find significant differences in the order of preference allotted to figure 1 (2º) by the three groups of evaluators, but did find significant differences in the order of preference for figure 2 (0º) (p=0.00). The Mann-Whitney test showed significant differences (p=0.001) in order of preference allotted to image 2 between orthodontists and laypeople. For figure 3 (4º) the Kruskal Wallis test p-value fell just within the limits of statistical significance (p=0.046) but no significant differences were found in order of preference for figure 3 (4º) between the three evaluator groups when the Mann Whitney test was applied with Bonferroni correction (p>0.016) ( Table 2).

**Table 2:**
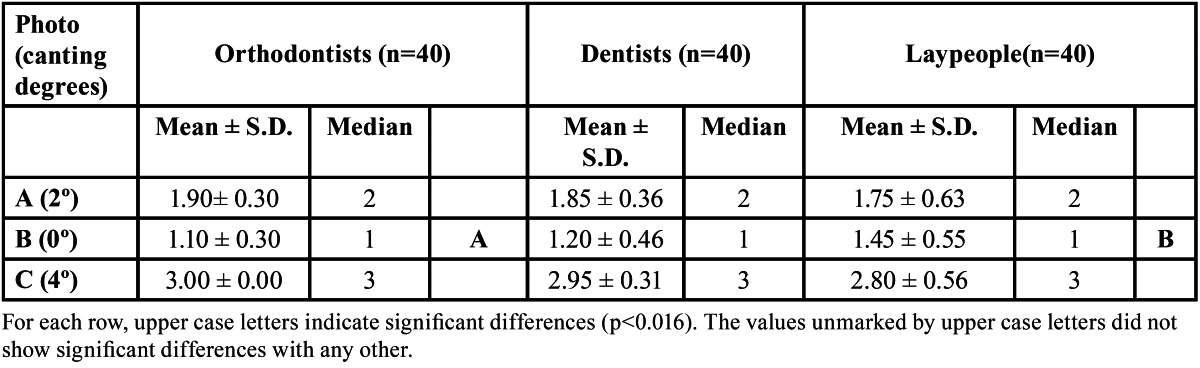
Mean and standard deviation (S.D.) for the order of preference given to each image.

The Levene test indicated that for the three images the standard deviation for the order of preference allotted by laypersons was significantly greater (Fig. 1 (2º) p= 0.00, Fig. 2 (0º) p= 0.00 and Fig. 3 (4º) p=0.00) than that of professionals (orthodontists and dentists).

## Discussion

The objective of this study was to determine how occlusal plane canting influences the esthetic assessment of the smile by orthodontists, general dentists and laypeople.

Various authors (13-16) suggest that there are differences between the perception of soft tissue and the perception of skeletal characteristics of patients with facial asymmetry, a fact that suggests that diagnosis should not be based solely on cephalometric features. For this reason, subjective evaluations of facial symmetry are important for performing correct diagnoses (17).

As in previous studies (3,10,12), the images used in this study were trimmed down to include the mouth alone in order to eliminate other features that might confuse perceptions,.

For quantifying subjective esthetic evaluations of the smile, some authors (8,10,18,19) have used a visual analogue scale (VAS). However, as in research carried out by Abu Alhaija et al. (2), the present study evaluated smiles using different rating scores (1=esthetically acceptable, 2=moderately acceptable and 3=esthetically unacceptable) as this method would produce simple, rapid and reproducible results.

All three groups evaluated the image manipulated to create a 2º occlusal cant (Fig. 1) as esthetically acceptable. These results coincide with other research that has observed that occlusal canting is not perceived by laypersons unless it exceeds 2º (20) or 3º (21). Indeed, Kokich et al. (3) found that laypersons did not detect this type of asymmetry unless it reached a 4º inclination.

The results of the present study indicate that all the evaluators (orthodontists, general dentists and laypersons) were sensitive to 4º occlusal canting. Nevertheless, orthodontists were less permissive with the 4º cant than general dentists and laypersons, as the former classed this as unacceptable while the latter groups evaluated it as moderately acceptable.

Meanwhile, Padwa et al. (22) have shown that occlusal canting greater than 4º is detected clinically with a frequency of over 90% by both professionals (trained in this field of observation) and laypersons (untrained in this field). However, Ker et al. (23) observed that laypersons found occlusal canting of up to 4º acceptable and a third of them found this acceptable up to a maximum of 6º.

With regard to the order of preference awarded to each photo, although all three groups put figure 2 (0º) in first place, orthodontists placed it in this position with greater frequency than laypersons. All put figure 1 (2º) in second place and figure 3 (4º) in third place. Orthodontists placed figure 3 in third place without exception. In other words, orthodontists were unanimous in finding figure 3 unacceptable.

Professionals coincided to a greater extent in their evaluations of the order of preference than non-professionals, as the standard deviation among laypersons was significantly greater. This shows that laypeople were the group who least coincided in placing the images in order of preference.

These results show that laypeople and general dentists find occlusal plane canting more acceptable than orthodontists. In this way, according to the present study, the profession of the evaluators affected the evaluation of smile esthetics when a canted occlusal plane was present. This is contrary to the findings of Padwa et al. (22) who suggest that in clinical examination dif-ferences in the detection of occlusal pane canting depend on the degree of inclination and not necessarily on the level of experience of the observers, the evaluations of professionals being similar to those of non-professionals.
